# Incidentally Detected Asymptomatic Epiglottic Capillary Hemangioma in a 66-Year-Old Female: A Case Report

**DOI:** 10.7759/cureus.104283

**Published:** 2026-02-26

**Authors:** Yu Kawasumi, Atsunobu Tsunoda, Seiya Yamaguchi, Yutaka Ozaki, Toshiharu Matsumoto

**Affiliations:** 1 Otolaryngology - Head and Neck Surgery, Juntendo University Nerima Hospital, Tokyo, JPN; 2 Diagnostic Radiology, Juntendo University Nerima Hospital, Tokyo, JPN; 3 Diagnostic Pathology, Juntendo University Nerima Hospital, Tokyo, JPN

**Keywords:** capillary hemangioma, dyspnea, endoscopy, epiglottis, larynx, magnetic resonance imaging

## Abstract

Laryngeal hemangiomas are benign vascular tumors that occur predominantly in children. Occurrence in adults is rare, particularly when developing in the epiglottis. This report describes a rare case of a pedunculated capillary hemangioma of the epiglottis in a 66-year-old female. The lesion was incidentally discovered during general anesthesia and was completely asymptomatic. Laryngeal endoscopy revealed a bluish-red, pedunculated mass with prominent vascularity, and MRI findings were consistent with a superficial vascular lesion without evidence of infiltration. Due to the potential risk of airway obstruction, surgical excision was performed with meticulous hemostasis. Histopathology confirmed a capillary hemangioma, and no recurrence was observed at one-year follow-up. This report highlights the importance of careful diagnostic evaluation and proactive surgical management of epiglottic vascular lesions in adults, even when asymptomatic. When surgical excision is clinically indicated, it allows for definitive histopathological diagnosis and eliminates the risk of potentially fatal airway obstruction.

## Introduction

Laryngeal hemangiomas are benign vascular tumors of unknown etiology that predominantly occur in children, whereas their occurrence in adults is uncommon [1-7]. These lesions are typically asymptomatic and are often found incidentally during routine examinations [2,7]. Among these, capillary hemangiomas of the epiglottis are extremely rare, with adult cases limited to isolated reports in the literature [8]. Because of their bleeding tendency and the critical anatomical location, determining the optimal treatment approach can be challenging [2,5,7]. In this report, we present a case of an incidentally detected, asymptomatic pedunculated capillary hemangioma at the epiglottic tip, highlighting its potential risks and management considerations.

## Case presentation

**Figure 1:**
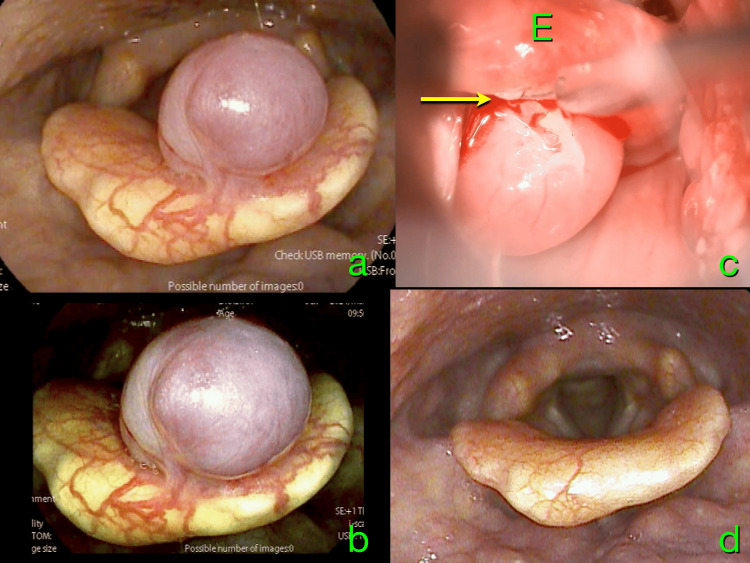
Laryngeal findings A bluish-red, pedunculated mass protruding into the laryngeal cavity from the tip of the epiglottis with prominent vascular engorgement on the surface of the epiglottis (a). These findings were clearly shown on vascular-enhanced imaging, indicating abundant blood flow (b). During surgery, the pedicle of this mass (arrow) was clearly visualized, then carefully coagulated and cut (c). A year later, no tumor was observed, and vascular engorgements on the surface of the epiglottis became less prominent (d)

A 66-year-old woman was incidentally discovered to have a tumor-like lesion on the epiglottis during general anesthesia for a partial mastectomy. Her postoperative course was uneventful, and she was referred to our department for further evaluation. At her initial presentation, she reported no symptoms, including hoarseness, dysphagia, throat discomfort, or hemoptysis. Laryngeal endoscopy revealed a bluish-red, pedunculated mass extending into the laryngeal cavity from the tip of the epiglottis (Figure 1a). The mucosal surface showed prominent vascular engorgement, suggesting abundant blood flow. These findings were more distinctly illustrated through vascular-enhanced imaging (Figure 1b).

**Figure 2:**
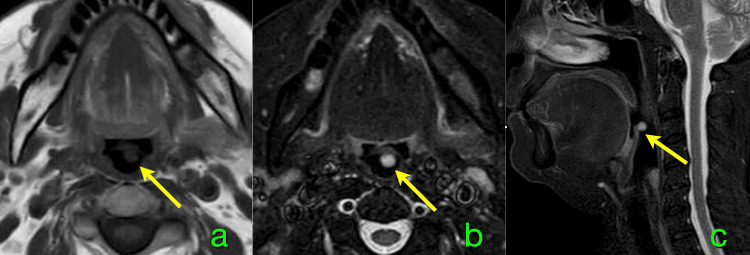
MRI images of this patient This lesion (arrow) shows low-signal intensity on axial T1-weighted image (a), high-signal intensity on axial (b) and sagittal (c) short tau inversion recovery images. This lesion does not infiltrate the epiglottis and is limited to the superficial layer (c) MRI: magnetic resonance imaging

MRI revealed a nodular lesion approximately 6 mm in diameter, attached to the epiglottis (Figure 2). The lesion exhibited low signal intensity on T1-weighted images, high signal intensity on short tau inversion recovery (STIR) images, and no abnormal signal on diffusion-weighted imaging (not shown). These findings suggested that the lesion was confined to the superficial layer of the epiglottis without evidence of infiltration. Given its location and relative frequency, an epiglottic cyst was initially suspected; however, the endoscopic appearance of the mass did not correspond to that of a typical epiglottic cyst.

**Figure 3:**
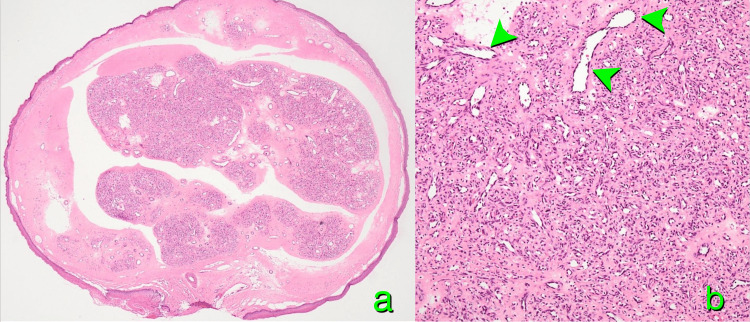
Histopathology A well-defined nodular lesion is observed in the submucosa. The tumor exhibits a lobulated structure, with diffusely dense capillary-like formations within the lesion. No evidence of invasion into surrounding tissue is noted, and the lesion demonstrates benign histological characteristics (hematoxylin and eosin, x20: a). The vascular lumens are lined by a single layer of flattened to cuboidal endothelial cells. This vascular lesion is observed throughout the entire lesion and is confined to the superficial tissue. This finding is clearly visible in the slightly dilated vein indicated by the arrowhead. No nuclear atypia or mitotic figures are observed. Erythrocyte accumulation is present within the vascular lumens, with mild thrombus formation noted in some areas (x100, b)

Despite the absence of symptoms, the mass exhibited pedunculated features and protruded into the laryngeal cavity, raising concerns about potential progressive enlargement and airway obstruction. Consequently, surgical excision was considered the optimal approach to prevent a potentially fatal outcome. Under general anesthesia, tracheal intubation was safely performed using a video laryngoscope. After inserting a direct laryngoscope, the mass and its pedicle were clearly visualized. The pedicle was meticulously coagulated to prevent bleeding into the airway. Subsequently, the pedicle was carefully dissected, and the mass was removed en bloc (Figure 1c). Intravenous hydrocortisone, 100 mg, was administered to prevent laryngeal edema. The postoperative course was uneventful, and the patient was discharged on the third postoperative day. Histopathological examination revealed lobulated and honeycomb-like proliferation of capillary-sized vessels in the central portion of the lesion (Figures 3a, 3b).

Based on these findings, the lesion was diagnosed as a capillary hemangioma. One year later, the patient was observed to be disease-free, and the vascular engorgement on the surface of the epiglottis had become less prominent (Figure 1d).

## Discussion

Hemangioma is a benign vascular tumor that predominantly occurs in children, especially infants [1]. It is observed in approximately 2.5% of neonates, with most cases developing before the age of two [1]. Around 60% of hemangiomas arise in the head and neck region [2]. According to the International Society for the Study of Vascular Anomalies (ISSVA) classification, vascular anomalies are broadly divided into vascular tumors and vascular malformations [3]. Hemangiomas fall under vascular tumors, and pediatric hemangiomas are further classified as congenital or infantile based on their clinical behavior [3]. In the laryngeal region, infantile hemangiomas and venous malformations are the most frequently reported [4]. Due to the anatomical characteristics of this area, such lesions can lead to upper airway obstruction, hemoptysis, hoarseness, and dysphagia, requiring careful management [4,5].

The occurrence of hemangiomas in adults is rare [6-9], and lesions confined to the epiglottis are extremely uncommon, with only isolated reports in the literature [8]. Ahmad et al. described cases of epiglottic capillary hemangioma with notable similarities to ours. Like our case, their patient was asymptomatic, but the tumor had a broad base and larger dimensions, requiring surgical removal to prevent airway obstruction. In the present case, although the tumor was smaller, it was pedunculated and located within the laryngeal inlet, posing a potential risk to respiratory function. Therefore, surgical excision is strongly advised for epiglottic hemangiomas. Potential etiological factors, including smoking, vocal overuse, endotracheal intubation, and laryngeal trauma, have been proposed [10]; however, cases such as the present one, without identifiable risk factors, suggest that the exact etiology remains unclear.

In adults, the diagnosis of laryngeal hemangioma primarily depends on endoscopic evaluation and imaging studies. In this case, prominent vascular engorgement around the tumor pedicle suggested abundant blood flow, indicating a vascular lesion. MRI is also valuable for assessing vascular lesions [11]. Characteristic radiological features of hemangiomas include low or isointense signals on T1-weighted images and high signal intensity on T2-weighted images [11]. However, lesions confined to the epiglottis require differentiation from cystic lesions and malignant tumors. Although gadolinium-enhanced MRI was not performed in this patient, it can be highly useful for establishing a definitive diagnosis of such vascular lesions [11].

Tumors arising in the epiglottis may affect swallowing and phonation, making careful evaluation of the lesion's characteristics essential, regardless of symptom presence. Regarding treatment, meticulous intraoperative hemostasis and postoperative edema management are crucial to ensuring surgical safety, as failure to do so could result in fatal dyspnea. In the present case, although the lesion was relatively small and asymptomatic, early surgical intervention was recommended due to the potential risk of airway obstruction.

Treatment strategies are determined by the size, location, and symptoms of the lesion. Reported surgical options include excision with bipolar cauterization [6], embolization and ultrasonic surgery [12], CO₂ laser resection [10], sclerotherapy [13], sclerotherapy combined with CO₂ laser resection [9], and argon plasma coagulation [14], with the modality chosen according to the lesion’s characteristics. In the case of a hemangioma with a pedicle, initial management of the pedicle appears to be a rational approach for achieving bleeding control [15,16]. In the present case, the tumor had a well-defined, localized pedicle, allowing for safe en bloc excision following electrofulguration. For broad-based or hemorrhagic lesions, the use of lasers or adjunctive hemostatic devices should be considered.

## Conclusions

Although adult epiglottic capillary hemangiomas may be incidentally detected and asymptomatic, they carry a potential risk of airway obstruction. Because capillary hemangiomas can mimic cystic lesions on MRI, careful endoscopic evaluation is essential. Management should be individualized, taking into consideration the lesion’s morphology, diagnostic uncertainty, and the risk of hemorrhage or airway compromise. When clinically indicated, surgical excision offers both a definitive diagnosis and curative treatment, with attention to adequate hemostasis and control of postoperative edema.

## References

[1] Zheng JW, Zhou Q, Yang XJ (2010). Treatment guideline for hemangiomas and vascular malformations of the head and neck. Head Neck.

[2] Martins RH, Lima Neto AC, Semenzate G, Lapate R (2006). Laryngeal hemangioma. Braz J Otorhinolaryngol.

[3] Wassef M, Blei F, Adams D (2015). Vascular anomalies classification: recommendations from the International Society for the Study of Vascular Anomalies. Pediatrics.

[4] Parhizkar N, Manning SC, Inglis AF Jr, Finn LS, Chen EY, Perkins JA (2011). How airway venous malformations differ from airway infantile hemangiomas. Arch Otolaryngol Head Neck Surg.

[5] Kiho L, Byard RW (2015). Acute fatal upper airway obstruction from an occult cavernous hemangioma of the larynx. J Forensic Sci.

[6] Mulliken JB, Fishman SJ, Burrows PE (2000). Vascular anomalies. Curr Probl Surg.

[7] Shukla TS (2022). Recurrence of laryngeal hemangioma in an adult: a case report. Cureus.

[8] Ahmad SA, Basit A, Javed S (2024). Epiglottic capillary hemangioma in an adult female: a rare case report. Int J Surg Case Rep.

[9] Kulkarni S, Dwivedi G, Singh A, Tiwari V (2023). Hemangioma - a rare cause of laryngeal growth in an adult and its management with sclerotherapy and laser surgery: a case report. Indian J Otolaryngol Head Neck Surg.

[10] Kimmelman CP, Sugar JO, Lowry LD (1979). Resident's page. Pathologic quiz case 2. Hemangioma of the vocal cord. Arch Otolaryngol.

[11] Abdel Razek AA, Elmokadem AH, Soliman M, Mukherji SK (2022). MR imaging of vascular malformations and tumors of head and neck. Magn Reson Imaging Clin N Am.

[12] Cipolla F, Ragusa M, Andaloro C, Bonanno A, Grillo C, Basile A, Mantia I (2020). Superselective embolization and transoral ultrasonic surgery of laryngeal hemangioma: a case report. B-Ent.

[13] Kamijo A, Hatsushika K, Kanemaru S, Moriyama M, Kase Y, Masuyama K (2013). Five adult laryngeal venous malformation cases treated effectively with sclerotherapy. Laryngoscope.

[14] Sharifi A, Nazemieh M, Moghadaszadeh M (2014). Supraglottic hemangioma as a rare cause of recurrent hemoptysis: a new treatment modality with Argon plasma coagulation (APC). Tanaffos.

[15] Chen IHK, Abdul Jalal SY, Singh B, Mat Baki M (2020). Adult laryngeal hemangioma - a rare case report. IMJM.

[16] Long X, Li Z, Liu Y, Zhen H (2024). Clinical application of low-temperature plasma radiofrequency in the treatment of hemangioma in nasal cavity. Pharynx and Larynx. Ear Nose Throat J.

